# Supplementary material for: RNA structure prediction using positive and negative evolutionary information
Source: PLoS Comput Biol. 2020 Oct 30;16(10):e1008387. doi: 10.1371/journal.pcbi.1008387 (PMC7657543; doi:10.1371/journal.pcbi.1008387)
Supplement: S1 File — Includes alignments, and source code. (GZ) [file pcbi.1008387.s008.tar.gz › supplemental_material/results/Figures/RF01750_ZMP-ZTP.5btp.R2R.sto.pdf]

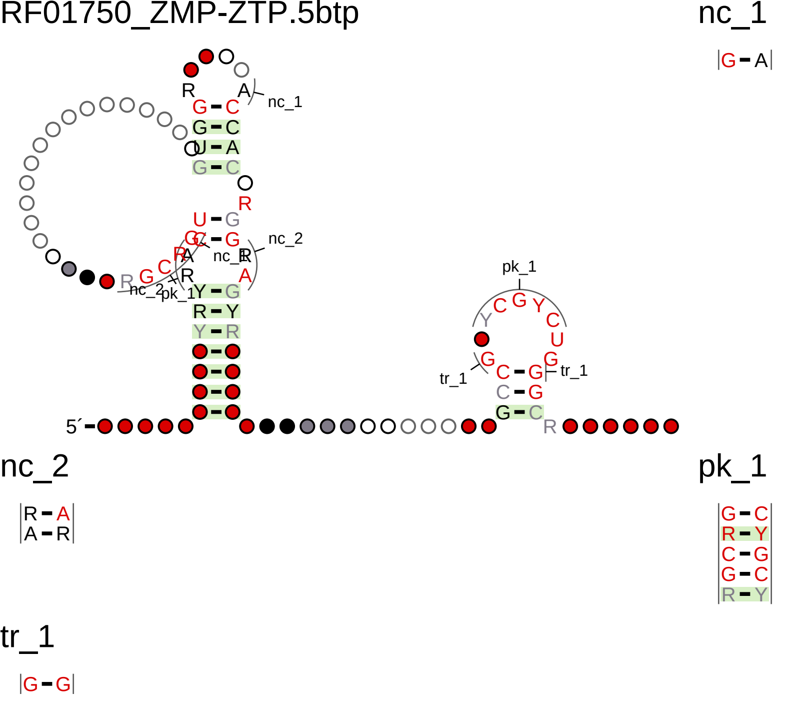

RF01750_ZMP-ZTP.5btp
nc_1
G A
nc_2
R A
A R
pk_1
G C
R Y
C G
G C
R Y
tr_1
G G
nc_1
nc_2
pk_1
tr_1
5´
